# A Tribute to the Memory of Dr. William Warren Potter by the New York State Board of Medical Examiners

**Published:** 1911-06

**Authors:** 


### A Tribute to the Memory of Dr. William Warren Potter by the New York State Board of Medical Examiners

THE death of William Warren Potter, M.D., the first and only president of the State Board of Medical Examiners, created under the laws of 1907, and for ten years previous thereto president of the State Board of Medical Examiners representing the Medical Society of the State of New York, severs one of the last links in the chain which connects the official medical surveillance of the practice of medicine in the state with those through whose efforts was created the law supervising the control of professional practice in New York. Dr. Potter's death was not unexpected; nevertheless it came as a great shock to those who for so many years were associated with him on the board and following so closely on the sudden death of Dr. Willis G. Macdonald and the loss of Dr. William S. Ely, it was a sore blow.

Dr. Potter was born and lived in a medical atmosphere. His father and his paternal grandfather were both practitioners of medicine and others of the family were likewise scions of the profession. He embarked in the actual study of his profession fresh from scholastic work and earned his medical spurs in Buffalo, the city of his adoption, in 1859. Engaged in practice with one of his uncles in Cowlesville, N. Y., when the civil war began, he early sought to lend his efforts to the government and became a factor in organising a local regiment which marched to the front in September, 1861, and Dr. Potter was its assistant surgeon. His activities were unceasing until, with his regiment, near the close of the war, he was mustered out of service. He was continuously advanced and at the war's close, by pronouncement of both the President and the Governor of New York State, he was brevetted lieutenant colonel for meritorious service. The 49th N. Y. volunteers and the 57th N. Y. volunteers were participants in most of the activities of the war and, with the exception of a short time during which he was a prisoner in Libby prison (to be exchanged under the earliest cartel) Dr. Potter was continuously in service with them. After the war he served as coroner of the District of Columbia for a short time and also became a general examining surgeon for the pension department of the governmental service. Craving for a practitioner's life, he resigned both of the above commissions and repaired to Mount Morris, Livingston County, thence to Batavia, Genesee County, where he practised his profession for a number of years during which time he was physician to the N. Y. State Institution for the Blind.

In 1881 he returned to Buffalo to practise general medicine but developed a strong leaning for gynecology and obstetrics. He was one of the pioneers in disassociating gynecologic work from general practice and helped to form the American Association of Obstetricians and Gynecologists of which organisation he was the only secretary and continuous editor of its transactions during a period of twenty-two years. He filled many other medical positions of trust and of honor. He was in the front rank of those who successfully fought for the abandonment of the old code of ethics in the Medical Society of the State of New York, of which latter body he was also president in 1891.

When the Medical Practice Act of 1890 went into effect, September 1, 1891, he was nominated by the State Medical Society for membership to the Board of Medical Examiners and the nomination was ratified by the Regents, his name being second on the list of appointees. When the board organised, his name was suggested for presidency, but after the first ballot, he asked that his friends vote for Dr. Wey, who was thereupon unanimously selected for that office. On the death of Dr. Wey, in 1897, Dr. Potter, who had continuously served as member of the board, was unanimously elected to the presidency. Each succeeding year he was elected and when the old board was supplanted by the present board, in keeping with the Medical Practice Act of 1907, he was reappointed examiner in obstetrics and gynecology, and then made president of the board, and so continued to the time of his death. He was an ideal presiding officer, thoroughly schooled in parliamentary tactics and ever watchful of the duties reposed in him. By virtue of his official functions, he was frequently called upon to act as presiding judge in cases where charges had been lodged with the board seeking to revoke the medical license of some offender against the law. His attitude on such occasions was highly judicial and he commanded at once the respect of lawyers and witnesses and secured the acclaim and admiration of his confreres on the board.

Dr. Potter's position as editor of the BUFFALO MEDICAL JOURNAL made it necessary for him to keep abreast of the times on all matters pertaining to sanitation and hygiene, health board exactions, the laws of the various states governing the practice of medicine, courses of study in medical and post-graduate schools, new developments in the science and practice of medicine, and in fact in all that was worth while in the profession. Punctilious in his regard for the exactions of the English language, his editorial utterances on all subjects relating to the topics which engaged his pen, were couched in selected language, excellently well phrased. In his later years he practically abandoned the actual practice of medicine in order to devote himself to the academic work connected with his official positions and to the preparation of the JOURNAL. He took the largest interest in the work of the Board of Medical Examiners, feeling a sense of personal pride in helping to enforce the laws which he had so strenuously advocated and which finally became effective in 1891.

Dr. Potter was well known to all of the men of prominence in the American medical profession, and it was not an infrequent occurrence to find him the center of a group of such at the close of a scientific meeting, relating in detail his recollection of some incident of the civil war, or recounting the salient features of a medico-legal controversy. His remarkably retentive memory coupled with great fluency of speech when deeply interested in a narration, made him a welcome companion at post prandial functions; added to this was the fact, recognised by all who knew him, that he was an appreciative listener. He was heroic in his sufferings, and when required, as on several occasions, he went to the operating table as a patient, with all the fortitude of a fatalist.

He was warmly attached to his associates on the board and seemed to feel a fatherly pride in each and all of them, and was delighted at any achievement accomplished by them. They in turn were loyal to him, and were proud to have him as their presiding officer. Each year he was selected as the board's representative to the annual meetings of the Council on Medical Education of the American Medical Association and each year he sat as the board's delegate in the sessions of the National Confederation of State Medical Examining and Licensing Boards, which latter organisation he helped to found, and of which he was president for a number of years.

Dr. Potter married when he was 21 years of age, and is survived by two daughters. His only son, Dr. Frank Hamilton Potter, a much-beloved young man, died in 1891.

No more worthy tribute could be paid to Dr. Potter, than to make a statement of the fact that he was true to his every trust, loyal to his friends, a tried servant of the state, strenuous in behalf of the best interests of the profession, a lovable companion, and a manly man.

We, the undersigned members of the State Board of Medical Examiners, in sorrow at his death, herewith attach our signatures to this memorial to Dr. William Warren Potter.

